# Developing As and Cu Tissue Residue Thresholds to Attain the Good Ecological Status of Rivers in Mining Areas

**DOI:** 10.1007/s00244-022-00915-w

**Published:** 2022-03-04

**Authors:** Iñigo Moreno-Ocio, Leire Méndez-Fernández, Maite Martínez-Madrid, Noemí Costas, Isabel Pardo, Pilar Rodriguez

**Affiliations:** 1 Department Zoology and Animal Cell Biology, University of the Basque Country, UPV/EHU, Box 644, 48080 Bilbao, Spain; 2 Department Genetics, Physical Anthropology and Animal Physiology, University of the Basque Country, UPV/EHU, Bilbao, Spain; 3 Department Ecology and Animal Biology, University of Vigo, Vigo, Spain

## Abstract

The study was performed on residue-effects datasets from polluted and unpolluted sites in the Nalón River basin (northern Spain). The effects were measured in terms of alteration of field macroinvertebrate communities, and measured as ecological status scores, and number of families and abundance of Ephemeroptera, Plecoptera and Trichoptera (EPT). Non-linear regression models of the field-measured tissue residues in 10 taxa related to the ecological status of the macroinvertebrate communities were used to derive effective tissue residues (ERs). These were estimated for the good/moderate boundary defined by the ecological quality ratio (EQRs) score and for the 50% reduction of EQR and EPT metrics. As, Cu, Hg and Se ERs were calculated for several macroinvertebrate taxa with different feeding styles. The ER dataset allowed us to estimate As and Cu hazardous concentrations (HC), using species sensitivity distribution models, and were interpreted as community thresholds. Further studies for Hg and Se are needed to complete the database required for HC estimation. The reliability and differences of the several thresholds were tested in a risk assessment using a tissue-residue approach (TRA) conducted with field organisms from Cauxa Creek, a tributary from the same basin exposed to high levels of metals in the sediments due to gold mining activities. This risk assessment identified that As and Cu tissue residues satisfactorily explained the reduction in the ecological status of the macroinvertebrate assemblages. Our results indicate that TRA can help in setting future environmental quality standards for the protection of aquatic biota.

**Graphical Abstract:**

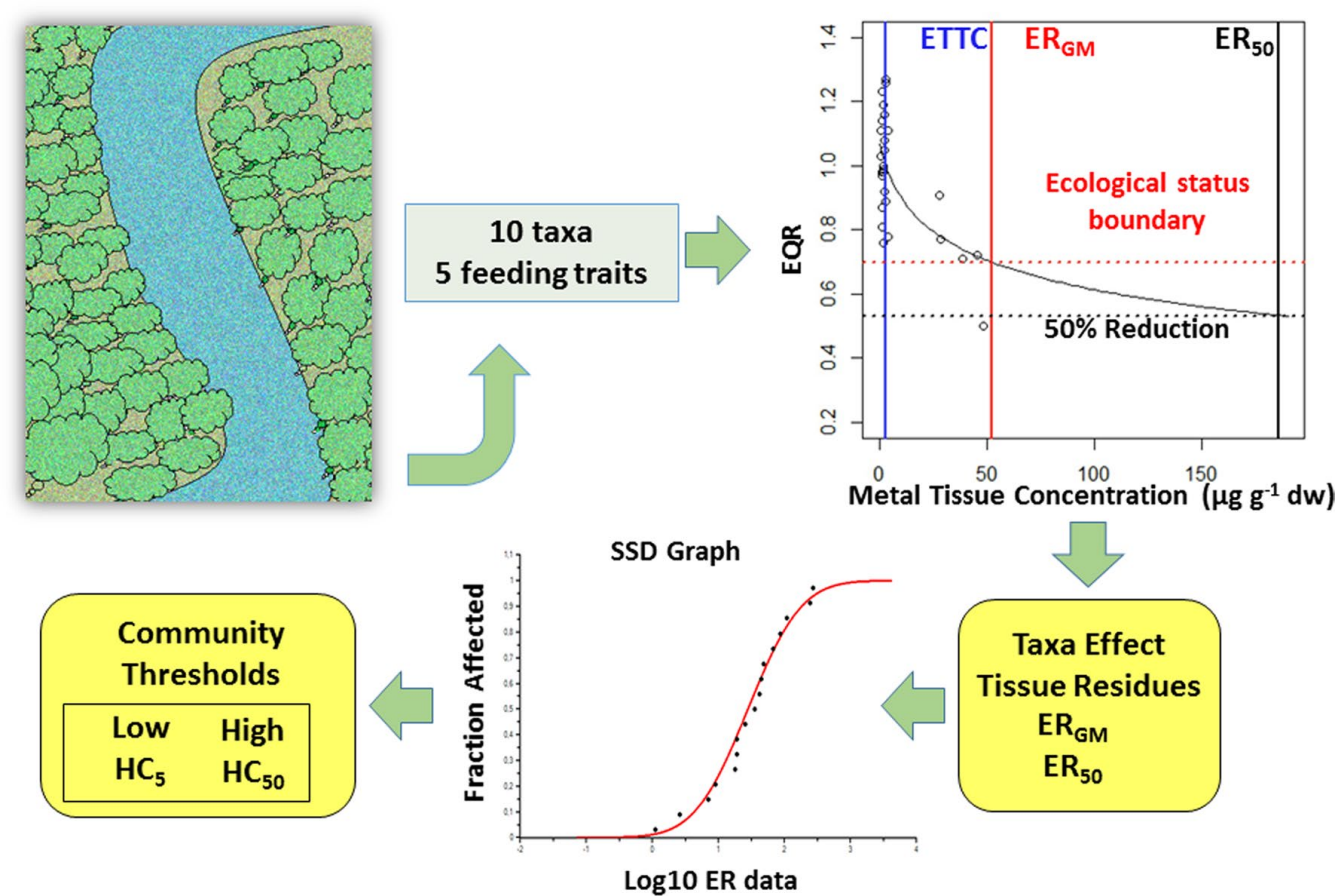

**Supplementary Information:**

The online version contains supplementary material available at 10.1007/s00244-022-00915-w.

Areas having a high level of metals and metalloids (hereinafter, metals) due to their lithology have often, historically been exploited for metal extraction. Mining activities usually result in the disposal of soil tailings in the areas adjacent to the mine, which can leach metals to rivers and cause alterations to aquatic communities in the absence of adequate management (Loredo et al. 2010). Monitoring the levels of contaminants in water is a common strategy in European countries, but this may be not enough to achieve the desired level of protection of field communities (EC 2008). For this reason, European policy also considers an important issue to set environmental quality standards in sediment and biota for some priority metals (Cd, Ni, Hg and Pb and their compounds) (EC 2008, Annex II). The objective of this environmental policy includes monitoring the bioaccumulation potential and bioassessment of impacts and trends for certain chemicals, thus ensuring protection against secondary poisoning at the community level. The measurement of metal tissue residues reflects bioavailability, thus reducing uncertainty about the actual bioavailable fraction of chemicals based on external concentrations (Sappington et al. 2011). There is also a general consensus on the convenience of including bioaccumulation data measured in indigenous organisms for sediment risk assessment (Chapman 2007; Adams et al. 2011) and for the integration of toxicological and ecological information toward regulatory applications using a tissue residue approach (McCarty et al. 2011). This integrative approach is especially suitable for assessing water quality in mining districts, where aquatic macroinvertebrates can accumulate high levels of metals (Cain et al. 2004; Solà et al. 2004; Méndez-Fernández et al. 2015) and can become a significant source of dietary uptake for their predators (Clements 1991; EC 2011). Furthermore, bioaccumulation can also be the cause of alterations in macroinvertebrate assemblages (Luoma et al. 2010; De Jonge et al. 2013; Bervoets et al. 2016).

The measurement of field alterations in the macroinvertebrate assemblage composition and structure is an essential component of the integrated assessment of ecological status, as established by the Water Framework Directive (EC 2000). In that context, the relationships of metal tissue residues in selected biomonitors to adverse effects on aquatic communities can be critical for developing reliable environmental quality criteria for the protection of biota. In a previous publication, the baseline metal concentration in 10 taxa was calculated from unaltered, field reference sites in the Nalón River basin (northern Spain) (Rodriguez et al. 2018), providing an estimate of the tissue concentration threshold for nine metals, below which alterations in the macroinvertebrate assemblages of the study region are unlikely (ETTC, ecological threshold tissue concentration). In the present study, we aimed to develop high-threshold tissue concentrations above which the impairment of macroinvertebrate assemblages is likely to occur. The impairment was measured through ecologically relevant metrics to protect field populations in the study region.

The tissue residue thresholds for benthic macroinvertebrates are valuable tools in water quality assessments in areas affected by mining activities since they can improve water and sediment quality standards; they can be used as screening benchmarks and provide necessary information in a weight-of-evidence approach using chemistry, toxicity and bioaccumulation data (Meador et al. 2014). We used two approaches to derive tissue residue thresholds: First, we derived the effective tissue residues (ERs) using nonlinear regression models relating taxa tissue residues against two general benchmarks: (1) the good/moderate boundary for macroinvertebrate ecological status, and (2) the 50% reduction of scores and metrics used to assess biological integrity of the macroinvertebrate assemblages. Second, using taxa-specific ERs estimated for As and Cu, we calculated the metal hazard concentrations (HCs) from a multitaxa risk assessment approach. Finally, the reliability of the proposed metal thresholds was tested in the Cauxa Creek risk assessment using a tissue-residue approach with field organisms.

## Material and Methods

### Study Area and Sampling

The Nalón River basin is located in northern Spain, and its catchment has experienced intense historical and present mining activity (Ordóñez et al. 2013). Samples of sediment and benthic macroinvertebrate taxa were collected and analyzed in two sampling campaigns, 14 reference sites in July 2014 and September 2015 and 15 test sites in September 2015, in the Nalón River basin. Sites were located at four macroinvertebrate community-based river types in Spain (R-T21, R-T25, R-T28, R-T31: MAGRAMA 2015). Detailed data on geographic information, water physicochemistry, and metal concentration in sediments and macroinvertebrate sampling strategy and composition from the study sites were published by Costas et al. (2018) (Online Appendix A, Table A1). Cauxa Creek is a small branch of the Narcea River, the main tributary of the Nalón River, and it is subject to active gold mining (Online Appendix A, Figure A1). Cauxa Creek was resampled in July 2016 for a followup risk assessment. Four sites were scrutinized, one upstream (P1) and three downstream from the gold mining effluents (from P2, closer to the mining effluents, to P4).

At each site, a composite sample of the upper sediment layer was obtained from fine deposits in the riverbed to measure the sediment metal concentration. Sampling of macroinvertebrates to evaluate bioaccumulation followed a river transect or multihabitat schema to collect 10 biomonitor taxa grouped into general functional feeding groups: scrapers (Baetidae and Heptageniidae), filterers (Ephemeridae, Hydropsychidae and Simuliidae), generalists (Ephemerellidae), predators (Perlidae and Rhyacophilidae) and deposit feeders (Lumbricidae and Microdriles oligochaetes) at each site (Table A1). Three field replicates consisting of 1–20 individuals of the larger size class were taken for tissue residue analysis. Detailed information on macroinvertebrate sampling procedures can be found in Rodriguez et al. (2018) and Table A1.

### Macroinvertebrate Metrics and Scores

The field community metrics were EPT richness (number of families of Ephemeroptera, Plecoptera and Trichoptera, EPT Fam), EPT abundance (number of individuals of EPT, EPT Ab), and the alteration in the ecological status of the macroinvertebrate assemblages, assessed through ecological quality ratios (EQRs), which are calculated as a quotient between the observed/reference value of a biological metric or score in a previously defined water body type (EC 2000). The EQRs were calculated for the river-type specific multimetric index (METI: MAGRAMA 2015) and for the scores derived from the NORThern Spain Indicators predictive model (NORTI: Pardo et al. 2014), both used in the study region and called METI-EQR and NORTI-EQR, respectively. Data on the tissue residues and site ecological status from the sampling campaigns of 2014 and 2015 in the Nalón River basin were incorporated into the regression models, while data from 2016 in Cauxa Creek were analyzed separately for a tissue-residue risk assessment.

### Metal Analysis in Sediments and Macroinvertebrate Tissue Residues

A total of nine metals (As, Cd, Cr, Cu, Hg, Ni, Pb, Se and Zn) were measured in sediments and biota. Limits of quantitation (LOQ) for biota and sediment are shown in Online Appendix A, Table A2. Values below the LOQ were replaced with ½ of LOQ for statistical analysis (US EPA 2000). In present study, we have assessed only four metals (As, Cu, Hg and Se) in biota that were relevant for the study area. All analytical methods can be examined in Costas et al. (2018) and Rodriguez et al. (2018) and are summarized in Table A1. All data in this study are reported in µg g^−1^ dw, and for data in the literature given on a ww basis, we used a conversion factor of 0.2 for all insect taxa (Meador 2011) and 0.1 for oligochaetes (Méndez-Fernández et al. 2013). For each taxon and site, tissue residues are given as the mean of 3 field replicates (on a few occasions, a single pooled sample was measured because of the scarcity of specimens).

### Data Interpretation and Statistical Analyses

**Fig. 1:**
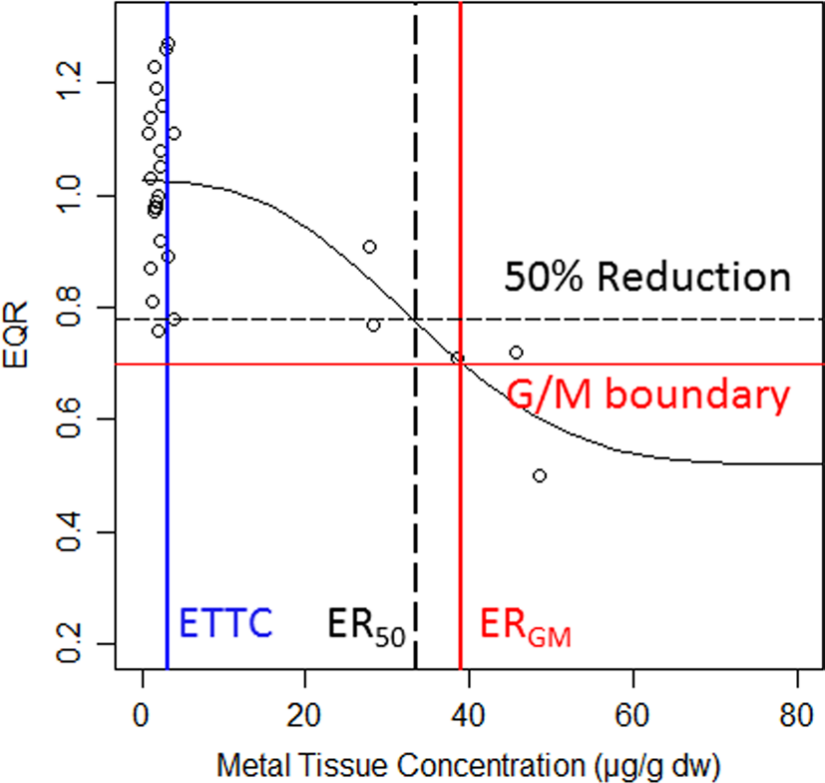
Scheme of the construction of the dose—response models between tissue concentration of the metal and the EQR (circles). The blue line is the ETTC value; the red lines are the EQR = 0.700 change point between Good and Moderate ecological status (G/M boundary) and the corresponding effective tissue concentration (ER_GM_). Superimposed are the black lines that represent the effective tissue concentration (ER_50_) related to the 50% reduction of the EPT indexes

Sediment metal concentration was assessed using the probable effect concentration (PEC: MacDonald et al. 2000) and the sediment pollution score (SedPoll: Costas et al. 2018) calculated from As, Cd, Cu, Hg, Pb and Se sediment concentrations in the Nalón River basin. In our previous contributions we described the baseline concentrations (Ecological Tissue threshold concentration, ETTC of 9 metals in several biomonitors of the Nalón River basin, Rodriguez et al. 2018), and more recently a bioaccumulation risk assessment was done, using the number of times the baseline concentrations in selected biomonitor taxa were exceeded (Rodriguez et al. 2021). In the present contribution, ERs were estimated on the same dataset for each taxon and metal from nonlinear regression models of the tissue residues (abscissa) vs EQRs (ordinate). Using the official cutoff for good/moderate ecological status (EQR = 0.700, EQR-ER_GM_: effective tissue residue above which the community status changes from good to moderate), a boundary intercalibrated for METI-EQR by the Central/Baltic group for benthic macroinvertebrate fauna (EC 2013a; MAGRAMA 2015). The effect on ecological status was also measured as a 50% reduction in the maximum EQR values (EQR-ER_50_: effective tissue residue above which a 50% reduction in the maximum EQR value occurs) in the model and a 50% reduction in the EPT richness and abundance metrics (EPT-ER_50_: effective tissue residue above which a 50% reduction in EPT richness or abundance occurs) (Fig. 1). In the regression models, ER_GM_ was calculated as the inverse function of the regression equations and solved numerically using Wolfram Mathematica 12 software from EQR = 0.700. The EQR-ER_50_ was estimated from the same selected models.

Nonlinear regression analyses were conducted using R software and the extension package drc (Ritz and Streibig 2005). After a preliminary analysis, the best fitted models were selected from a set of 6 commonly used sigmoid models (log-logistic and Weibull) with 3 and 4 parameters:

$${\text{Log}} - {\text{logistic }}\left( {{\text{LL4}}} \right):y = c + (d - c)/\left( {1 + \exp \left( {b\left( {{\text{Log }}x - {\text{Log}} e} \right)} \right)} \right)$$

$${\text{ Weibull }}1 \, \left( {W1.4} \right):y = c + \left( {d - c} \right)\exp \left( { - \exp \left( {b\left( {{\text{Log}} x - {\text{Log}} e} \right)} \right)} \right)$$

$${\text{Weibull 2 }}\left( {{\text{W2}}.{4}} \right):y = c + \left( {d - c} \right)\left( {1 - \exp \left( { - \exp \left( {b\left( {{\text{Log}} x - {\text{Log}} e} \right)} \right)} \right)} \right)$$

The parameters *c* and *d* are the lower and upper asymptotes for the *y* variable, respectively, and they are in the same units as the *y* variable; parameter *e* is the inflection point of the dose–response curve and provides the ER_50_ value in the log-logistic models; and parameter *b* is proportional to the slope of the dose–response curve at dose *e* (Ritz 2010). Three-parameter models were obtained from each model when *c* = 0.

The models with a difference in Akaike’s Information Criteria (AIC) < 2 were selected (Burnham and Anderson 2002) and were validated when: (1) the *c* parameter was ≥ 0, the minimum value of the EQR and macroinvertebrate assemblage metrics; (2) the estimated ER was within the field range of tissue residue values; and (3) the standard error of the estimated ER was lower than the ER value. In the case of regression models built with the EQRs, we also considered for the validity of the model that the *d* parameter was ≤ 1.4, the maximum of the EQR value (Pardo et al. 2010). Graphical tests of the standardized and studentized residuals for the selected equations were examined, and studentized residuals were always <|3|.

For each taxon and metal, several EQR-ER_GM_, EQR-ER_50_ and EPT-ER_50_ values were estimated from the validated models, and then averaged. The effective tissue residues (ER) estimated on the 50% reduction in the scores have the advantage over the ER_GM_ that we have been able to calculate the 95% confidence limits of the estimates. EQR-ER_50_ and ER_GM_ should be similar since the good/moderate boundary of 0.700 is half the maximum expected EQR. Using species sensitivity distribution (SSD) models (ETX v.2.1 program, Van Vlaardingen et al. 2004), the 5th and 50th percentile hazard concentrations (HC_5_, HC_50_) for the macroinvertebrate assemblages were calculated using the taxa ER_GM_, EQR-ER_50_ and EPT-ER_50_ mean values.

Finally, the aforementioned threshold values were used in a risk assessment of Cauxa Creek, using the average ratios of the field tissue residues (TRs) to the EQR-ER_GM_ for all the biomonitor taxa present at each site. Four quality classes were considered for the risk assessment based on a tissue-residue approach: (1) Low risk for the community when TR/ER ≤ 1, (2) Moderate risk when TR/ER = 1.1–2.0, (3) High risk when TR/ER = 2.1–10.0, and (4) Very High risk when TR/ER > 10. The same classification was used to assess the ratios of TR to the HC_50_ values. No risk was expected only when the mean tissue residues were < HC_5_ or the ETTC (ecological threshold tissue concentration).

## Results

### Dose–Response Models and Effective Tissue Residues for As, Cu, Hg and Se in Macroinvertebrates

Dose—response models for the relationship of field taxa tissue residues to the macroinvertebrate assemblage metrics (METI- and NORTI-EQRs and EPT richness and abundance) were built when possible for 10 taxa. Tissue residues uploaded to the models from 15 potentially polluted sites from the Nalón River basin are shown in Online Appendix B, Table B1; data from 14 reference sites were reported by Rodriguez et al. (2018). In the study area, METI-EQR values ranged from 0.50–1.27, and NORTI-EQRs ranged from 0.26–1.27. The maximum EPT richness varied from 6 to 25 families, and the EPT abundance varied from 96 to 7333 individuals per site (2.5 m^2^). A total of 254 (out of 960 calculated) dose–response models were validated following the criteria reported in “Material and Methods”Section; 87 models for As, 75 for Cu, 52 for Se, and 40 for Hg. ER_GM_ was calculated from 128 models (64 models using METI-EQR data and 64 models using NORTI-EQRs), and the EQR-ER_50_ was calculated from 86 models (Online Appendix B, Table B2); EPT Fam-ER_50_ was estimated from 58 regression models, and EPT Ab-ER_50_ was estimated from 68 models (Online Appendix B, Table B3). Overall, ERs were calculated for all the study taxa from several selected models: Microdrile oligochaetes (42 models), Rhyacophilidae (33), Baetidae and Heptageniidae (28 each), Lumbricidae (26), Hydropsychidae (23), Ephemerellidae and Ephemeridae (22 each), Simuliidae (19) and Perlidae (11).

The mean ERs calculated for each metal and taxon relative to the good/moderate ecological status boundary (ER_GM_) are shown in Table 1. The ER_GM_ concentrations estimated from NORTI-EQRs were generally 1–3 times lower than the ER_GM_ concentrations derived from the METI-EQRs. The highest As ER_GM_ values were for Simuliidae or Microdrile oligochaetes, while the lowest values were found in the predators Rhyacophilidae and Perlidae. The Cu ER_GM_ was higher for Heptageniidae and Ephemerellidae, while the deposit feeders and filterers had lower values. Only a small number of models were validated for Hg and Se.

**Table 1:** Mean effective tissue residues (ERs) (µg g^−1^ dw) calculated for As, Cu, Hg and Se from non-linear regression models of the tissue concentration and the EQR for each site and taxon

	Mean METI–EQR versus TR regression models			Mean NORTI–EQR versus TR regression models		
Taxon	EQR–ER_50_ (range)	EQR–ER_GM_ (range)	Mean ER_GM_/ETTC	EQR–ER_50_ (range)	EQR–ER_GM_ (range)	Mean ER_GM_/ETTC
As						
Baetidae	71.0 (32.1–186)	41.6 (37.3–52.2)	13.4	99.5 (51.5–123)	18.9 (16.3–21.7)	6.1
Ephemerellidae	–	67.7 (66.7–68.7)	10.5	–	35.5	5.5
Ephemeridae	–	–	–	–	44.3	6.9
Heptageniidae	–	48.6 (47.6–49.4)	7.7	7.8 (7.6–8.0)	9.0 (8.0–10.8)	2.3
Hydropsychidae	16.1 (2.6–29.5)	–	–	–	25.5 (23.1–27.8)	12.7
Lumbricidae	17.2	19.1	1.4	84.9 (83.5–86.2)	85.7 (84.5–86.9)	6.2
Microdrile	561	243.1 (230–256)	16.8	542.4 (509–570)	109.4 (79.0–126)	7.6
Rhyacophilidae	11.1 (10.7–11.7)	7.1 (6.9–7.4)	4.2	1.1	1.1	1.5
Perlidae	–	–	–	–	2.6	1.5
Simuliidae	120	269 (196–341)	59.8	–	17.8	4.0
Cu						
Baetidae	39.2	104.5	3.6	33.6 (32.8–34.6)	38.1 (36.6–39.6)	1.3
Ephemerellidae	237 (236–240)	194 (191–199)	6.5	–	134.4	1.5
Ephemeridae	37.0 (36.5–37.8)	–	–	19.1 (16.2–21.0)	22.9 (16.5–27.3)	1.4
Heptageniidae	220 (199–241)	409.7 (407–414)	5.1	98.1 (97–100)	158 (156–160)	2.0
Hydropsychidae	–	–	–	15.6 (15.3–15.9)	18.5 (17.9–19.1)	1.2
Lumbricidae	146 (145–147)	82.4 (77.7–86.8)	7.1	208	45.7	4.0
Microdrile	36.0 (35.7–36.4)	37.7 (37.3–38.1)	1.8	31.6 (30.1–33.0)	30.9 (29.6–32.2)	1.5
Perlidae	118 (105–131)	98.5 (97.7–99.3)	2.9	–	–	–
Rhyacophilidae	67.1	51.2 (49.5–52.5)	2.6	32.2 (31.9–32.5)	39.4 (32.9–44.7)	2.0
Simuliidae	–	–	–	15.8 (15.5–16.1)	37.2 (36.4–37.9)	0.7
Hg						
Baetidae	–	3.6	13.7	–	–	–
Ephemeridae	0.05	–	–	0.10	–	–
Heptageniidae	–	–	–	0.06 (0.06–0.07)	–	–
Perlidae	–	–	–	0.07 (0.06–0.07)	–	–
Rhyacophilidae	0.60	0.59	1.2	0.23 (0.21–0.24)	0.35 (0.34–0.36)	0.7
Simuliidae	–	7.9 (7.8–8.0)	13.1	–	0.97 (0.96–0.97)	1.6
Se						
Baetidae	–	20.1 (18.7–22.1)	1.8	15.2 (14.8–15.6)	8.7 (8.6–8.7)	0.7
Heptageniidae	–	–	–	3.4 (3.2–3.5)	–	–
Hydropsychidae	3.0	–	–	1.1 (0.9–1.1)	–	–
Microdrile	7.1 (6.6–7.6)	16.8	2.2	5.8	5.9	0.8

Their ranges are given when *n* > 1. EQR-ER_50_ was calculated as the tissue residues corresponding to a 50% reduction in the EQR score. EQR-ER_GM_ was estimated from the models for the official EQR value used as the boundary between good and moderate ecological status of the macroinvertebrate assemblages. Ratios of the ER_GM_ to the baseline ETTC are shown. ETTC, ecological threshold tissue concentration; TR, tissue residues

Regarding the ratios of the ER_GM_ to the baseline ETTC for each metal and taxon (Table 1), the METI-ER_GM_ values were usually 4–20 times the ETTC for As (but up to 60 for Simuliidae); these ratios ranged 2–7 times for Cu, 1–14 for Hg, and approximately 2 for Se. The ratios of NORTI-ER_GM_ to ETTC were generally lower, 2–13 for As, and varied typically from 1–2 for Cu, Hg and Se. In most instances, the EQR-ER_50_ values ranged between 0.4 and 7.1 times (mean = 1.7) the corresponding ER_GM_.

**Fig. 2:**
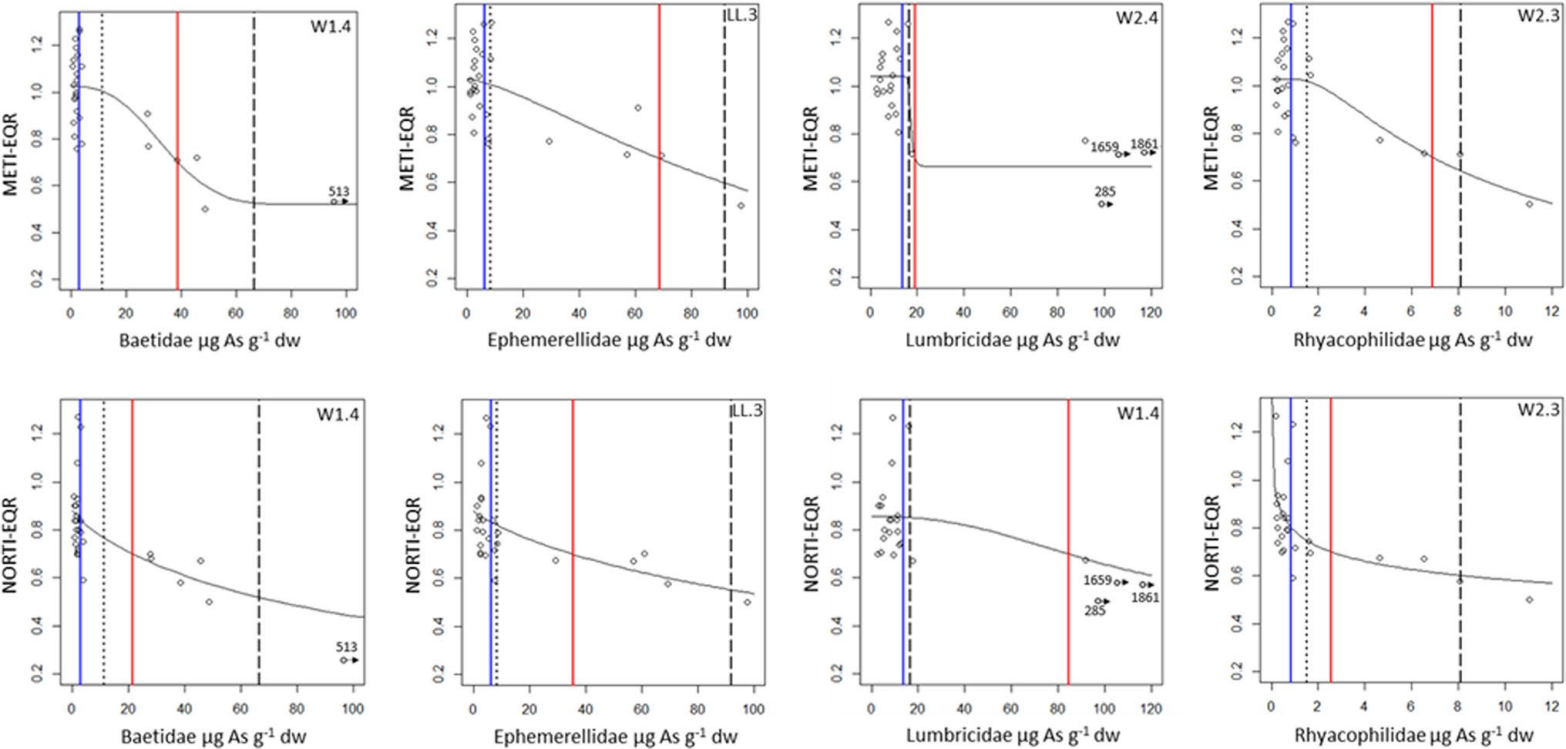
Arsenic dose—response models for METI- and NORTI-EQRs calculated for several taxa (Baetidae, Ephemerellidae, Lumbricidae and Rhyacophilidae) selected from sites in the Nalón River basin. Superimposed lines mark the low and high threshold values. The low threshold is in all instances the baseline concentration in the basin (ETTC), in blue. There are several parameters useful as high thresholds: METI- and NORTI-ER_GM_ in red. For comparison, the ER_50_ values calculated when possible for the 50% reduction of EPT number of families and abundances are also superimposed in black-dashed line and in black-dotted line, respectively. The represented regression models are indicated on the upper right corner of each plot

**Fig. 3:**
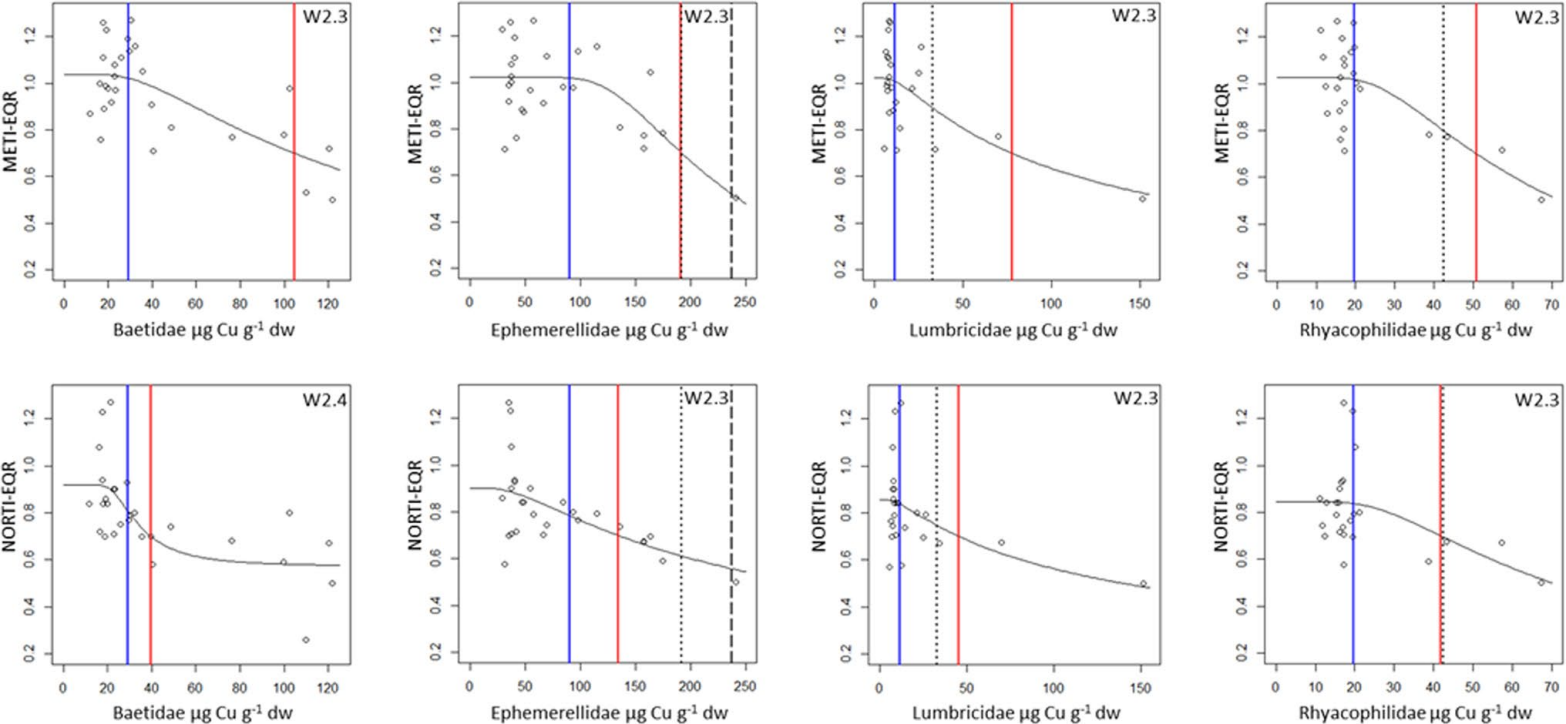
Copper dose—response models for METI- and NORTI-EQRs calculated for several taxa (Baetidae, Ephemerellidae, Lumbricidae and Rhyacophilidae) selected from sites in the Nalón River basin. Superimposed lines mark the low and high threshold values. The low threshold is in all instances the baseline concentration in the basin (ETTC), in blue. There are several parameters useful as high thresholds: METI- and NORTI-ER_GM_ in red. For comparison, the ER_50_ values calculated when possible for the 50% reduction of EPT number of families and abundances are also superimposed in black-dashed line and in black-dotted line, respectively. The represented regression models are indicated on the upper right corner of each plot

We found EPT Fam-ER_50_ for As (Table 2) to be lower for EPT abundance than for EPT richness. This is interpreted as As tissue residues causing a reduction in abundance of sensitive taxa before having an effect on the number of families. For Cu, the ratios of Cu EPT-ER_50_ to ETTC were 1–3 (except for Rhyacophilidae, with ratios < 1), with similar values for richness and abundance. In the case of Hg and Se, the EPT-ER_50_ estimates were limited to a few taxa and, in most instances, < 1 µg g^−1^ for Hg, which resulted in ratios to ETTC ≤ 1. The EPT Ab-ER_50_ was 3 and 2 times higher than the baseline ETTC for As and Cu, respectively (Table 2; Figs. 2 and 3, for Baetidae, Ephemerellidae, Lumbricidae and Rhyacophilidae). However, EPT Fam-ER_50_ values were much higher than ETTC for As (mean ratio 16.9) but only 2 times higher for Cu and equal or lower for Hg and Se (Table 2). The ratios for Se were very variable, although based on a limited number of data.

**Table 2:** Effective tissue residues (ER_50_) of As, Cu, Hg and Se (µg g^−1^ dw) related to the 50% reduction in the EPT number of families (EPT Fam) and the EPT abundance (EPT Ab)

	As		Cu		Hg		Se	
Taxon	EPT Fam–ER_50_	EPT Ab–ER_50_	EPT Fam–ER_50_	EPT Ab–ER_50_	EPT Fam–ER_50_	EPT Ab–ER_50_	EPT Fam–ER_50_	EPT Ab–ER_50_
Baetidae	24.1	11.4	–	–	–	–	19.5	5.7
Ephemerellidae	92.1	8.4	237.2	192.0	0.06	–	–	–
Ephemeridae	20.5	–	38.0	–	0.14	0.61	–	–
Heptageniidae	8.1	18.1	–	–	–	–	–	4.8
Hydropsychidae	107.7	–	16.7	–	–	–	2.1	4.3
Lumbricidae	16.7	–	–	32.8	–	–	10.0	14.9
Microdrile oligochaetes	318.7	85.5	–	55.1	–	0.72	4.4	8.0
Perlidae	–	–	167.8	77.6	–	0.06	–	–
Rhyacophilidae	8.1	1.5	–	42.4	–	–	–	1.1
Simuliidae	171.5	8.4	–	–	–	0.72	–	–
ER_50_/ETTC ratio	1.2–53.9	1.3–5.9	1.1–2.7	0.3–2.8	0.2–0.3	0.0–1.2	0.5–1.6	0.2–1.1
Mean	16.9	2.9	2.2	2.1	0.25	1.0	0.8	0.7

ER_50_ are calculated as the mean ER_50_ values estimated per taxon from the validated regression models. Abbreviations: EPT, Ephemeroptera, Plecoptera and Trichoptera; ETTC, Ecological Threshold Tissue Concentration

Last, using the SSD models based on the taxa ERs, we estimated hazard concentrations for the integrity of the whole community. The HC_5_ and HC_50_ were estimated for As and Cu (Table 3) but not for Hg and Se due to the limited number of data points. The HC_5_ derived from the EQR-ER_GM_ values for both As and Cu were similar to those estimated using EQR-ER_50_ or EPT-ER_50_ data (Table 3). The HC_50_ values estimated from the same SSD models were 11–25 times higher than the corresponding HC_5_ for As and 4–5 times higher for Cu. The respective 90% confidence limits of HC_5_ and HC_50_ did not overlap, which supports their use as low and high community thresholds, respectively.

**Table 3:** Hazard concentrations (HC_5_, HC_50_) for As and Cu and their 90% confidence limits (CL) derived from SSD models using several effective tissue concentrations (ER)

Chemical	HC	EQR-ER_GM_ (90% CL)	*n*	EQR-ER_50_ (90% CL)	*n*	EPT-ER_50_ (90% CL)	*n*
As	HC_5_	2.41	17	1.67	11	2.12	15
		(0.74–5.27)		(0.20–5.74)		(0.59–4.82)	
	HC_50_	27.98		41.05		24.13	
		(15.06–51.97)		(14.62–115.3)		(12.50–46.58)	
	As ETTC-HC_50_	4.24 (2.46 –7.30)					
Cu	HC_5_	15.45	16	11.63	16	14.31	9
		(7.52–24.70)		(5.25–19.51)		(4.32–27.48)	
	HC_50_	64.62		56.40		67.43	
		(44.47–93.89)		(37.34–85.20)		(38.44–118.3)	
	Cu ETTC-HC_50_	29.16 (19.23–44.21)					

EQR-ER_GM_: Effective tissue residues related to the EQR boundary between Good and Moderate ecological status of the macroinvertebrate community; EQR-ER_50_: Effective tissue residues related to the 50% reduction in the EQR; EPT-ER_50_: Effective tissue residues related to the 50% reduction of the EPT metrics of abundance and richness. For comparison, the HC_50_ derived from the baseline concentrations of As and Cu in unpolluted reference sites is given (ETTC-HC_50_, Rodriguez et al. 2018). *n*: number of data included in each of the models

### Cauxa Creek Risk Assessment: A Tissue Residue Approach

The sediment metal concentration in the 2016 campaign in Cauxa Creek showed that 4 metals, As, Cu, Hg and Se, exceeded the PEC values in sites downstream of mine effluents (P2-P4) (Online Appendix B, Table B4). At P2, As was up to 48 times the PEC value, Cu 13 times and Se 4 times, while the Hg concentration barely exceeded the PEC. Additionally, sediment metal pollution assessed by the SedPoll index evaluated the upstream site, P1, as unpolluted or similar to the reference, while sites P2 to P4 were assessed as medium to highly polluted.

At site P1, ten biomonitors used in the Nalón River basin were found, but downstream (P2-P4), only four of them were present at the four study sites: Baetidae, Ephemerellidae, Lumbricidae and Rhyacophilidae. These taxa represent four different feeding styles: scraper, generalist, deposit feeder and predator. Bioaccumulation levels of As and Cu were high, up to a maximum of 49 times the ETTC (Baetidae) at P2, as expected from the high metal concentration in the sediment. However, the Hg and Se tissue residue to ETTC ratios were usually < 1, with a maximum of 1.9 for Hg and 1.6 for Se (Lumbricidae) in P2 (Online Appendix B, Table B5).

The Cauxa Creek risk assessment based on the tissue residue approach was performed using the average ratios of tissue residues to their corresponding high thresholds (EQR-ER_GM_) calculated for each taxon present (Table 4). These ratios were also averaged for each feeding style (Online Appendix B, Table B6). Site P1, upstream from the gold mine, showed in all cases low risk due to As, Cu, and Hg tissue residue ratios to EQR-ER_GM_. In all cases, downstream sites were assessed as High Risk related to As bioaccumulation (Table 4). The Cu bioaccumulation risk assessment result was variable, depending on the EQR-ER_GM_ used. Se tissue residues downstream of the mine were assessed as Low or Moderate Risk. The Hg showed Low Risk related to the METI and NORTI assessments. The importance in this risk assessment of the high ratios obtained for predators for As and Se in downstream sites can be seen in Table B6.

**Table 4:**
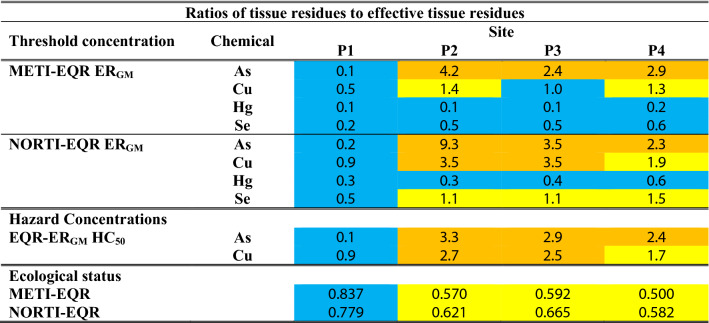
Cauxa river risk assessment based on the mean ratios of the tissue residues in biomonitor taxa to metal thresholds

The effective tissue residues (EQR-ER_GM_) and the community hazard concentration (HC_50_) were used as high thresholds. The ratios are classified within the following classes: Low Risk (≤ 1) in blue, Moderate Risk (1.1–2.0) in yellow, High Risk (2.1–10.0) in orange and Very High Risk (> 10) in red. The ecological status of the benthic macroinvertebrate assemblages in the four sites of the Cauxa River assessed by the METI and NORTI EQRs are shown in blue (Good) and yellow (Moderate)

Finally, the risk assessment for As and Cu tissue residues using their ratios to HC_50_ for the macroinvertebrate assemblage showed a consistent evaluation with the EQR-ER_GM_ assessment (Table 4). The upstream site (P1) was at Low Risk, while sites downstream of the mine effluents (P2-P4) were assessed from High to Moderate Risk, in parallel with a decreasing concentration of pollutants in the sediment and with decreasing ecological status score values, both for METI and NORTI (< 0.700, good/moderate boundary). When HC_5_ was used as the benchmark, the four sites showed some degree of risk due to As and Cu bioaccumulation (tissue residue ratios to HC_5_ > 1).

## Discussion

The main goal in water quality management is to attain or maintain the ecological integrity of aquatic communities, thus incorporating ecological realism into the regulatory framework (Kiffney and Clements 2002). In Spain, the evaluation of exposures to contaminants in biota is based on the *standstill* principle, which states that priority substances in the sediments or biota should not significantly increase their concentration on a long-term basis (MAGRAMA 2015). This evaluation is inadequate to prevent loss or to recover the good ecological status of aquatic communities, since bioaccumulation can be one of the causes of adverse effects, hindering the recovery of the good ecological status of the macroinvertebrate assemblages. In that context, the tissue residue approach provides a step in the evaluation of causal agents derived from contaminants (Meador et al. 2014) and is a necessary tool for developing tissue quality criteria that should improve risk assessment and remediation policies.

A comparison of our thresholds with others (calculated using different approaches, but also associated with a reduction in macroinvertebrate community metrics), showed that the similarity among values depends on the biomonitors selected. In other studies, adverse effects on aquatic organisms related to As bioaccumulation were similar to the As HC_50_ (28 µg g^−1^ dw) estimated in the present study: e.g. 1.3–5 µg As g^−1^ ww (≈ 6.5–25 µg g^−1^ dw) (Eisler 2000), and 6.6 µg As g^−1^ ww (≈ 33 µg g^−1^ dw) (DEQ 2007). Higher As thresholds were reported by Bervoets et al. (2016), but this probably was related to their selection of relatively tolerant biomonitors (Diptera: Chironomidae: 65–130 µg g^−1^ dw and tubificid oligochaetes: 85–93 µg g^−1^ dw). In our study, the filterer Simuliidae (Diptera) and sediment-feeder Microdrile oligochaetes also showed higher As ERs (Table 1).

The Cu ER_GM_ in the present study attained high values (155.7–414.1 µg g^−1^ dw for Heptageniidae and 134.4–199.3 for Ephemerellidae,Table B2). However, the ER_GM_ HC_50_ (65 µg Cu g^−1^ dw, Table 3) was similar to that proposed by Bervoets et al. (2016) (57 µg g^−1^ dw). Higher Cu ERs have been reported for specific biomonitors. For example, the value for Hydropsychidae (> 170 µg g^−1^ dw) was associated with a reduction or absence of heptageniids and ephemerellid mayflies (Rainbow et al. 2012), and the values for Heptageniidae (165.2–349.5 µg g^−1^ dw) were associated with a 20–50% loss in macroinvertebrate richness (De Jonge et al. 2013).

The range of values estimated for Hg ER_GM_ in the present study is wide (0.35–7.9 µg g^−1^ dw), but the higher values estimated for Simuliidae and Baetidae (Table 1) should be viewed with caution, since they are very biased with respect to the median value (0.97 µg g^−1^ dw). The lower range is comparable to the Hg guidelines proposed for the biota (e.g. 0.12–1.68 µg g^−1^ dw: CCME 2000; 20 ng g^−1^ ww ≈ 0.1 µg g^−1^ dw: EC 2013b).

Only a small number of Se ER_GM_ could be estimated in our study, ranging from 5.9–20.1 µg g^−1^ dw, comparable to the tissue residues associated with sublethal toxic effects reported by DeBruyn and Chapman (2007) (1–30 µg g^−1^ dw), and dietary Se thresholds for fish (e.g. 3 to 11 µg g^−1^ dw, May et al. 2008).

In the selection of suitable bioaccumulation thresholds, it is desirable that there is a clear but not very large gap between low and high thresholds to reduce the probability of false positives or negatives in the risk assessment. This was the case for most selected taxa, which showed an ER_GM_/ETTC ratios of > 1–10 for As and Cu (Table 1). However, for Hg and Se these ratios were calculated in very few instances. In the case of Se, the low ratios are probably associated with its essential nature for metabolism, and to the fact that most species of aquatic macroinvertebrates are relatively insensitive to Se (Janz et al. 2014). The database for Hg and Se should be completed with supplementary sites to better understand these low ratios and provide a better risk assessment in the future.

The estimated metal thresholds (ERs) vary by one or two order of magnitude, depending on the selected biomonitors. However, the metal Hazard Concentrations (HC_5_ and HC_50_) calculated from different biological effective tissue residues are much more comparable to each other. The HC values estimated from SSD models using EQR-ER_50_ and ER_GM_ were very similar, and also were similar to the ER_50_ calculated from the 50% reduction of the abundance and richness of the EPT taxa. This suggests that thresholds estimated from the good/moderate boundary are a reliable measure of ecological status. The HC_5_ 90% confidence limits of As and Cu calculated from different ERs overlapped with each other and with the confidence limits of the baseline ETTC-HC_50_ (Table 3), thus making the HC_5_ a reliable low threshold for risk assessment. Nevertheless, when tissue concentrations are close to HC_5,_ a comparison with the baseline concentrations of the biomonitor will improve the accuracy of the risk assessment. In addition, the accuracy of the HC thresholds would probably improve if the sensitivity of the biomonitors was within the HC confidence interval of the metal, avoiding false negatives or positives.

The risk assessment exercise in Cauxa Creek largely affected by gold mining clearly pointed toward the influence of As and Cu bioaccumulation by macroinvertebrates on the altered ecological status of sites downstream of the mine (P2-P4). The EQR-ER_GM_ thresholds are taxon specific, thus it is possible to get different assessments depending the biomonitors. In this case study, the average of the ratios of TR/ EQR-ER_GM_ from a selection of several biomonitor species comprise a wide range of sensitivity to the metals, which helps getting a weighted assessment of the risk. The risk assessment through NORTI-ER_GM_ was closer to the ecological status assessment than through METI-ER_GM_. However, in Cauxa Creek, the ratios of the tissue residues to the community high thresholds (HC_50_) resulted in a straightforward and consistent risk assessment, comparable to the ecological status assessment. The HC thresholds also have the advantage of being less dependent on the presence of certain biomonitor taxa at the study sites.

Despite the relevance of the interaction of several metals to evaluate the effects on the biota due to bioaccumulation, there are few studies that have addressed the effects of metal mixtures in field organisms (e.g., De Jonge et al. 2013). In the present study, the interactions of Se with other bioaccumulated trace metals (e.g., As, Cu and Hg) must be analyzed in more detail, since interactions have been demonstrated in the literature. In particular, Se is recognized for its potential in reducing the toxicity of Hg compounds (Hamilton 2004), an issue that requires further research in Cauxa Creek. Specific thresholds for the protection of higher levels in the aquatic trophic chain should also be developed in the future for the satisfactory protection of aquatic communities. This problem is complex since the risk of metal transfer from macroinvertebrates to aquatic wildlife depends on the diet specificity, prey availability, accumulation pattern and ability of the organisms to depurate the metals (Rainbow 2018).

## Conclusions

This study is the first to derive the effective tissue concentration from the cutoff value of good/moderate ecological status of the macroinvertebrate assemblages using ten biomonitor taxa. The ecological status of the field community is regularly evaluated by the water authorities, following the European water directive; therefore, EQR can be useful to calculate environmental thresholds for macroinvertebrates derived through a tissue residue approach. The models provide a complementary tool not only to monitor environmental risk due to bioaccumulation, but also to predict alterations in the ecological status of field macroinvertebrate assemblages. The HC_5_ and HC_50_ calculated for As and Cu are promising since they can be readily applicable as low and high thresholds in the mining districts of northern Spain. They can contribute to setting future environmental quality standards for the protection of aquatic biota. The same approach can be implemented in other European river basins to calculate threshold concentrations in the biota related to reductions in intercalibrated metrics of ecological status.

## Supplementary Information

Below is the link to the electronic supplementary material.

Supplementary file1 (DOCX 1171 KB)

Supplementary file2 (XLSX 56 KB)
