# Supplementary material for: Developing As and Cu Tissue Residue Thresholds to Attain the Good Ecological Status of Rivers in Mining Areas
Source: Arch Environ Contam Toxicol. 2022 Mar 4;82(3):379–90. doi: 10.1007/s00244-022-00915-w (PMC8971172; doi:10.1007/s00244-022-00915-w)
Supplement: Supplementary file 1 — Supplementary file1 (DOCX 1171 KB) [file 244_2022_915_MOESM1_ESM.docx]

**Table A1-** Sampling design and methods for metal analysis of sediment and macroinvertebrates and the evaluation of the ecological status of macroinvertebrate assemblages in the Nalón River basin.

| **SAMPLING DESIGN** | **METHODOLOGY** | **REFERENCES** |
| --- | --- | --- |
| Sediment | In every site, a 0.5 L of a composite sediment sample was collected with a stainless-steel spade from the upper 5**–**10 cm layer of fine sediment settled along an approximately 25-m reach of the riverbanks. Samples were taken to the laboratory on ice and stored at 4 °C in the dark. | Méndez-Fernández *et al*. (2015) |
| Macroinvertebrate Ecological Status | A total sampled area of 2.5 m^2^. At each study site, 20 **‘**sample units**’** distributed proportionately in the main habitats existing along a 100 m reach were combined. Kick-net (500 μm mesh). Fixed and preserved in 70% ethanol. | Spanish official protocol ML-Rv-I (2013)  Pardo *et al*. (2014) |
| Macroinvertebrate bioaccumulation | For each taxon, when possible 3 replicates consisting of 1–20 individuals were collected per site, placed in 15-ml tubes containing river water, and stored in ice. After 5-10 h, the macroinvertebrates were cleaned in dechlorinated water, identified and frozen at -20°C. | Rodriguez *et al*. (2018) |
| **SAMPLE ANALYSIS** | **METHODOLOGY** | **REFERENCES** |
| Sediment | After drying at room temperature, the sediment fraction < 63μm was digested using microwave extraction method, in concentrated nitric acid and concentrated hydrochloric acid (US EPA 3051 protocol). Metal analysis: ICP-MS (Agilent 7700X). Reference materials: Buffalo River sediment (RM8704, USA) and Sewage Sludge-3 (CRM 031-040, UK). | USEPA (2007) |
| Macroinvertebrate Ecological Status | Taxa richness and abundance were evaluated at the family level (except for aquatic oligochaetes and Hydracarina), under 57X magnification dissecting microscope (Olympus SZX9). | Costas *et al*. (2018) |
| Macroinvertebrate bioaccumulation | Samples were freeze-dried and weighted (Sartorius M3P balance). Acid digestion: 70% Nitric Acid Baker Instra-Analyzed+30% H_2_O_2_ Merck Suprapur. Metals analysis: ICP-MS (7500ce, Agilent Technologies). Reference material: NIST2976 mussel tissue. | Clements (1994)  Rodriguez *et al*. (2018) |

| **Funcional traits** | **Families or higher taxa** | **Genera, spp** |
| --- | --- | --- |
| Scraper (grazer) | Baetidae | Mostly *Baetis* spp |
|  | Heptageniidae | *Ecdyonurus, Heptagenia,* and *Epeorus* spp |
| Generalist | Ephemerellidae | *Serratella ignita* |
| Collector-filterer | Ephemeridae | *Ephemera* spp |
|  | Hydropsychidae | Mostly *Hydropsyche* spp |
|  | Simuliidae | undetermined |
| Collector-gatherer (deposit feeder) | Lumbricidae | *Eiseniella tetraedra* |
|  | Microdrile oligochaetes | Mostly Lumbriculidae (mainly *Stylodrilus heringianus)* |
| Predator | Rhyacophilidae | *Rhyacophila* spp |
|  | Perlidae | *Perla* sp*, Dinocras cephalotes* |


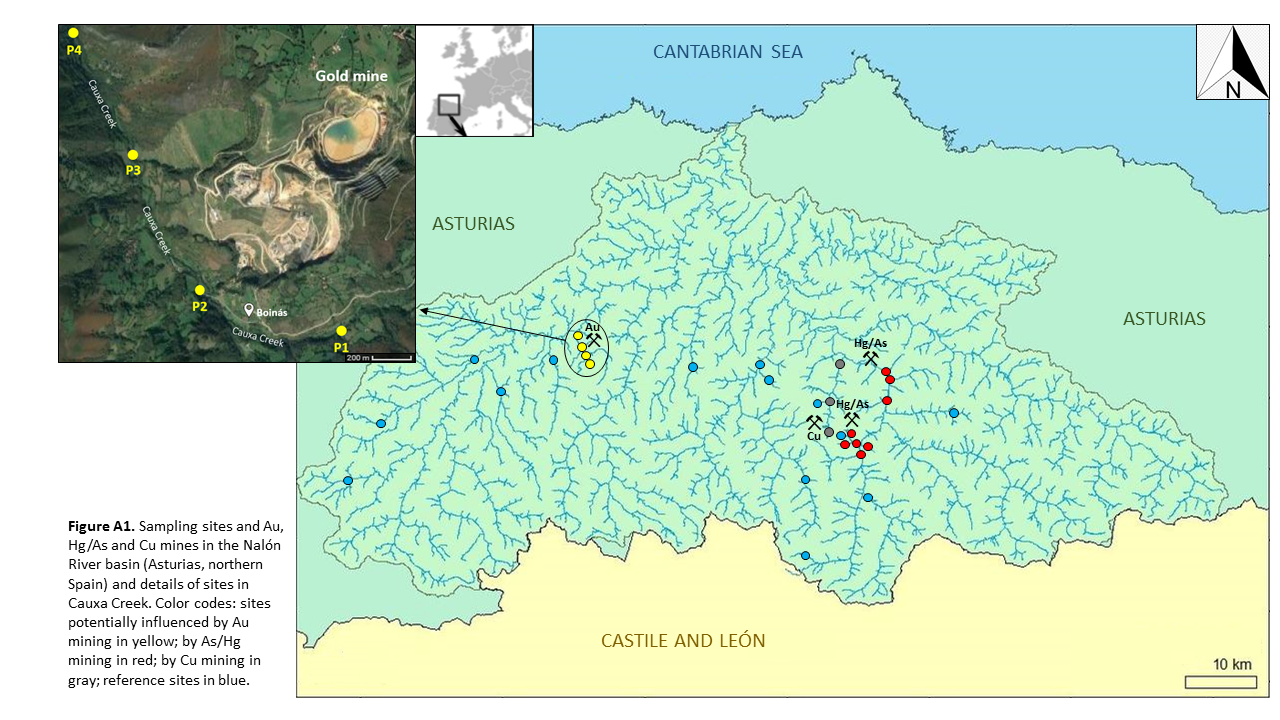


**Table A2-** Limits of quantitation (LOQ) of As, Cd, Cu, Cr, Hg, Ni, Pb, Se and Zn for tissue residues and sediment (µg l^-1^) from 2014, 2015 and 2016 sampling campaigns.

|  | **Year** | **As** | **Cd** | **Cu** | **Cr** | **Hg** | **Ni** | **Pb** | **Se** | **Zn** |
| --- | --- | --- | --- | --- | --- | --- | --- | --- | --- | --- |
| **Tissue** | 2014 | 0.009 | 0.014 | 0.060 | 0.040 | 0.014 | 0.073 | 0.040 | 0.567 | 0.833 |
|  | 2015 | 0.010 | 0.010 | 0.097 | 0.049 | 0.031 | 0.171 | 0.079 | 0.586 | 1.443 |
|  | 2016 | 0.050 | 0.008 | 0.197 | 0.040 | 0.008 | 0.196 | 0.070 | 0.520 | 0.740 |
| **Sediment** | 2016 | 0.010 | 0.010 | 0.100 | 0.030 | 0.010 | 0.030 | 0.030 | 0.400 | 50 |

**References A1**

Clements WH (1994) Integrated laboratory and field approach for assessing impacts of heavy metals at the Arkansas River, Colorado. *Environ Toxicol Chem* 13, 397–404.

Costas *et al*. (2018) Sensitivity of macroinvertebrate indicator taxa to metal gradients in mining areas in Northern Spain. *Ecol Ind* 93, 207–218.

Méndez-Fernández *et al*. (2015) Sediment toxicity and bioaccumulation assessment in abandoned copper and mercury mining areas of the Nalón River basin (Spain). *Arch Environ Contam Toxicol*, 68(1), 107-123.

ML-Rv-I, (2013) Protocolo de muestreo y laboratorio de fauna bentónica de invertebrados en ríos vadeables. Spanish Government, Ministry of Agriculture, Food and Environment (ed), 23 pp.

Pardo *et al*. (2014) An invertebrate predictive model (NORTI) for streams and rivers: sensitivity of the model in detecting stress gradients. *Ecol Ind* 45, 51–62.

Rodriguez *et al*. (2018) Baseline tissue levels of trace metals and metalloids to approach ecological threshold concentrations in aquatic macroinvertebrates. *Ecol Ind* 91, 395–409.

USEPA (2007) Method 3051A (SW-846): Microwave Assisted Acid Digestion of *Sediments*, Sludges, and Oils. Available online: <https://www.epa.gov/hw-sw846/sw-846-test-method-3051a-microwave-assisted-aciddigestion-sediments-sludges-soils-and-oils>
